# *BRAF* testing in metastatic colorectal carcinoma and novel, chemotherapy-free therapeutic options

**DOI:** 10.1007/s00292-021-00946-5

**Published:** 2021-07-14

**Authors:** Michael Hummel, Susanna Hegewisch-Becker, Jens H. L. Neumann, Arndt Vogel

**Affiliations:** 1 Institut für Pathologie der Charité, Universitätsmedizin, Campus Charité Mitte, Virchowweg 16/17a, 10117 Berlin, Germany; 2 Onkologische Schwerpunktpraxis, Facharztzentrum Eppendorf, Hamburg, Germany; 3 Pathologisches Institut der Medizinischen Fakultät, Ludwig-Maximilians-Universität München, Munich, Germany; 4 Klinik für Gastroenterologie, Hepatologie und Endokrinologie, Medizinische Hochschule Hannover, Hannover, Germany

**Keywords:** Cetuximab, Encorafenib, BRAF-inhibitors, Protein kinase inhibitors, Proto-oncogene proteins B‑raf, Cetuximab, Encorafenib, BRAF-Inhibitoren, Proteinkinaseinhibitoren, Protoonkogene B‑Raf-Proteine

## Abstract

In the past 25 years, treatment of metastatic colorectal cancer (mCRC) has undergone profound changes. The approval of newer chemotherapeutics such as irinotecan and oxaliplatin was followed in 2005 by the first targeted therapies, for example, monoclonal antibodies directed against the epidermal growth factor receptor (EGFR), as cetuximab and panitumumab, or the angiogenesis inhibitors bevacizumab, ramucirumab, and aflibercept. With the rapidly progressing molecular characterization of mCRC in the last 10 years and the classification of the disease in four consensus subtypes, further changes are emerging, which will promote, among other things, the introduction of protein-kinase inhibitors developed for specific molecular aberrations as well as immune checkpoint inhibitors into the treatment algorithm.

Thorough molecular pathologic testing is indispensable today for guideline-compliant treatment of mCRC patients. In addition to RAS testing as a precondition for the therapy decision with regard to cetuximab and panitumumab, *BRAF *testing is of considerable relevance to allow decision making with regard to the newly approved chemotherapy-free combination of the BRAF inhibitor encorafenib and cetuximab in cases where a *BRAF-V600E* mutation is detected. Additional diagnostic tests should also include genome instability (microsatellite instability). Overall, more and more molecular alterations need to be investigated simultaneously, so that the use of focused next-generation sequencing is increasingly recommended.

This overview describes the prognostic relevance of *BRAF *testing in the context of molecular pathologic diagnostics of mCRC, presents new treatment options for *BRAF*-mutated mCRC patients, and explains which modern DNA analytical and immunohistochemical methods are available to detect *BRAF *mutations in mCRC patients.

## Abstract (de)

Die Therapie des metastasierten kolorektalen Karzinoms (mKRK) hat in den letzten 25 Jahren tief greifende Veränderungen erfahren. Auf die Zulassung neuerer Chemotherapeutika folgten ab 2005 die ersten zielgerichteten Therapien, die sich gegen den epidermalen Wachstumsfaktorrezeptor (EGFR) bzw. gegen Rezeptoren vaskulärer endothelialer Wachstumsfaktoren (VEGFR) richteten. Mit der fortschreitenden molekularen Charakterisierung des mKRK in den letzten 10 Jahren und der Einteilung der Erkrankung in 4 Konsensus-Subtypen zeichnet sich weiterer Wandel ab, unter anderem durch Einführung speziell entwickelter Proteinkinaseinhibitoren wie auch Immuncheckpoint-Inhibitoren in den Therapiealgorithmus.

Eine angepasste molekularpathologische Testung ist heute für eine leitliniengerechte Behandlung von mKRK-Patienten unabdingbar. Neben der *RAS*-Testung als Voraussetzung für die Therapieentscheidung bezüglich Cetuximab und Panitumumab ist die *BRAF*-Testung äußerst relevant, um – im Falle des Nachweises einer *BRAF-V600E*-Mutation – eine Therapieentscheidung zugunsten der neu zugelassenen, chemotherapiefreien Kombination aus dem BRAF-Inhibitor Encorafenib und Cetuximab treffen zu können. Eine erweiterte Diagnostik sollte auch die Genominstabilität (Mikrosatelliten-Instabilität) einbeziehen. Insgesamt müssen immer mehr molekulare Alterationen simultan untersucht werden, sodass sich zunehmend die Verwendung des fokussierten Next Generation Sequencing empfiehlt.

Diese Übersichtsarbeit beschreibt die prognostische Relevanz der *BRAF*-Testung im Rahmen der molekularpathologischen Diagnostik des mKRK, stellt neue Therapieoptionen zur Behandlung *BRAF*-mutierter mKRK-Patienten vor und erläutert, welche modernen DNA-analytischen und immunohistochemischen Verfahren zur *BRAF*-Diagnostik von mKRK-Patienten zur Verfügung stehen.

In patients with colorectal cancer (CRC), *BRAF *testing together with *RAS *testing is an established component of molecular biological diagnostics before initiating first-line therapy according to guidelines. The aim of this review is to provide an up-to-date overview of the significance of *BRAF* as a prognostic and predictive biomarker, to show new therapeutic options for metastatic CRC (mCRC) patients with *BRAF *mutations, and to describe the currently available diagnostic methods for *BRAF *testing.

Despite significant advances in treatment, CRC continues to be one of the cancer entities with an unfavorable prognosis in Europe with about 250,000 deaths per year and an annual incidence of more than 500,000 new cases [1]. As the molecular characterization of metastatic CRC (mCRC) and the classification of CRC into molecular subtypes progresses [2], the number of options for the use of targeted therapies is also increasing, with molecular diagnostics becoming ever more important.

*BRAF *mutations are present in around 8–12% of patients with mCRC [3, 4]. More than 95% of all *BRAF *mutations are *BRAF*-*V600* mutations, where valine (V) is mostly substituted by glutamic acid (E) at position 1799 in codon 600 (in exon 15) of the *BRAF *gene. Apart from this most frequent mutation, *BRAF*^*V600E*^, there are also less common mutations in codon 600, in which valine at position 1799 is substituted by lysine (*BRAF*^*V600K*^), aspartic acid (*BRAF*^*V600D*^), methionine (*BRAF*^*V600M*^), or arginine (*BRAF*^*V600R*^) [5]. Clinically, a comparison of the *BRAF*-*V600* mutation with the significantly less common *BRAF *mutations in codons 594 and 596 shows that the former is more often found in right-sided and mucinous primary tumors with peritoneal metastasis, whereas BRAF^*594/596*^ tumors have a better prognosis [3]. Unless expressly described otherwise, all the statements made in the following sections of this paper refer to *BRAF*^*V600E*^ mutations in mCRC.

B‑Raf is a key kinase in the Ras/RAF/MEK-mitogen-activated protein kinase (MAPK) signaling pathway, which is involved in the regulation of cell growth. The alteration of the *BRAF *gene due to mutation leads to the constitutive activation of this protein kinase, thus causing uncontrolled cell division and consecutively leading to (neo-)angiogenesis and metastasis [6]. Resulting from studies on the CRC transcriptome, mCRC has been classified into four consensus subtypes (*consensus molecular subtypes*, CMS). *KRAS *mutations predominantly occur in the epithelial, “metabolic” subtype CMS3, which is characterized by metabolic dysregulation and partly also by chromosomal and microsatellite instability (MSI) [2, 7]. *BRAF *mutations, however, are often seen in the “MSI-immune” subtype CMS1, which is dominated by somatic hypermutation and MSI.

It is extremely rare that BRAF mutations occur together with a mutation of the *RAS *gene. According to the current German S3 guideline, molecular testing for the presence of both mutations should be carried out before initiating first-line therapy, wherever possible. In this context, *BRAF *testing should best be done simultaneously with the *RAS* test or sequentially after exclusion of the *RAS* mutation [3].

## Oncogenic properties of the *BRAF *mutation

*BRAF*, which is an oncogenic driver in mCRC patients, was already established as a therapeutic target structure in malignant melanoma many years ago [8]. Serrated adenomas of the intestine that are associated with a *BRAF *mutation show molecular, morphological, clinical, and epidemiological characteristics that differ from those of adenomas and which develop during a “classic adenoma-carcinoma sequence” based on mutations of the *APC *gene [9, 10]. The *BRAF*-driven form of sessile serrated adenomas (SSA) leads to impaired apoptosis of crypt epithelia followed by senescence with epigenetic promoter (CpG) methylation and decreased expression of various genes (e.g., h*MLH1, MGMT, p16*) [3, 9]. SSAs as a tumor pre-stage and precursor lesion are flat polyps that barely protrude from the mucosa, predominantly occur in the right-sided colon, and are difficult to detect even endoscopically [3, 9]. Patients with large SSA have a higher risk of developing colorectal cancer; women with SSA have a five-fold higher risk than men [3].

### *BRAF* mutation: a clearly negative prognostic factor in CRC

In mCRC, advanced age is a negative prognostic marker, as is tumor location proximal to the left flexure [11]. As part of an investigation on further potential prognostic markers in this indication, the influence of *BRAF *mutations and MSI on metastatic spread and prognosis was analyzed within a large retrospective case series: *BRAF*-mutant tumors, particularly those harboring a V600E mutation, are associated with a significantly poorer overall survival (OS) than *BRAF *wild-type tumors (median 10.4 versus 34.7 months; hazard ratio [HR] = 10.66, *p* < 0.001), as well as with a higher rate of peritoneal and distant lymph node metastasis [12]. The prognostically highly unfavorable impact of *BRAF*^*V600*^ was also repeatedly reported in randomized controlled trials [4, 13, 14]; a detailed discussion of the prognostic impact of *BRAF* mutations and their connection with microsatellite stability and instability as a further biomarker can be found in two recent reviews [6, 15]. Apart from hereditary non-polyposis-associated colorectal carcinoma (HNPCC), MSI occurs in mCRC patients with an estimated frequency of only 4–8% [4]. If *BRAF *mutations and MSI occur simultaneously—the frequency is reciprocally about one third each—these alterations constitute sporadic defects of mismatch repair (dMMR) [3, 4]. MSI patients appear to have a better prognosis than patients with microsatellite stability (MSS) [16]; although the number of published cases is still limited, the available clinical evidence suggests that patients with a *BRAF*^*V600E*^ mutation and MSS have a poorer outcome than those with *BRAF*^*V600E*^ and high MSI status. In the metastatic setting, the combination of *BRAF*^*V600E*^ and MSS seems to predominate, with the BRAF mutation determining the poor outcome [6, 13, 17, 18, 19, 20].

### *BRAF* mutation: unclear predictive value with regard to former conventional therapies

The predictive relevance of *BRAF *mutations for the use of anti-epidermal growth factor receptor (EGFR) therapy, i.e., the two monoclonal antibodies cetuximab and panitumumab, is currently under debate due to the fact that *BRAF* and *RAS *mutations are almost mutually exclusive [21] and that *RAS *mutations are known negative predictive factors for the use of anti-EGFR therapy. In CRC, cetuximab and panitumumab are approved for use in *RAS *wild-type patients only: for their use in patients with a *BRAF *mutation, only limited data are available from subgroup analyses of larger confirmatory studies (Table 1), as well as from retrospective case series derived from clinical routine data [22, 23].

**Table 1:** Studies and retrospective analyses on the significance of *BRAF* as a predictive marker in the use of anti-epidermal growth factor receptor therapies (A) and anti-vascular endothelial growth factor therapies (B) for *BRAF*-mutated metastatic colorectal cancer

Study/phase (or type) of study	Comparison	“Backbone” (therapy)	N_ITT_/N_BRAF-mut._ (if specified)^a^	BRAF assessment^b^ (diagnostic method)	OS (months)	PFS (months)	ORR (%)	HR [95% CI]	Reference
**A. Anti-EGFR therapies**									
*First-line therapy*									
**Crystal** + **OPUS**/III (R-SGA, pooled)	Cetuximab + CTx *vs*. CTx	FOLFIRI (Crystal), FOLFOX4 (OPUS)	1535/32 *vs*. 38	PCR (PNA and melting curve)	14.1 *vs*. 9.9	7.1 *vs*. 3.7	22 *vs*. 13	OS: 0.62 [0.36–1.06] PFS: 0.67 [0.34–1.29]	[36]
**PRIME**/III (R-SGA)	Panitumumab + CTx vs. CTx	FOLFOX	1183/24 *vs*. 29	PCR (Heteroduplex analysis)	10.5 *vs*. 9.2	6.1 *vs*. 5.4	NR	OS: 0.90 [0.46–1.76] PFS: 0.58 [0.29–1.15]	[37]
**FIRE‑3**/III (R-SGA)	Cetuximab + CTx vs. Bevacizumab + CTx	FOLFIRI	752/23 *vs*. 25	Pyrosequencing	12.3 *vs*. 13.7	6.6 *vs*. 6.6	52 *vs*. 40	OS: 0.79 [0.43–1.46] PFS: 0.84 [0.47–1.51]	[38]
*Second-line therapy*									
**20050181**/III (R-SGA)	Panitumumab + CTx vs. CTx	FOLFIRI	1186/22 *vs*. 23	PCR/Sanger	5.7 *vs*. 4.7	2.5 *vs*. 1.8	NR	NR	[39]
**PICCOLO**/III (R-SGA)	Panitumumab + CTx vs. BSC	Irinotecan	460/37 *vs*. 31	PCR/Pyrosequencing	NR	NR	11 *vs*. 6	NR	[40]
*Therapy-refractory patients (≥* *2 previous therapies)*									
**20020408**/III (R-SGA)	Panitumumab *vs*. BSC	∅	463/18^**c**^	PCR (sequencing)	NR	NR	0 (*vs*. 0)	NR PFS: 0.34 [0.09–1.24]	[41]
**CO.17**/III (R-SGA)	Cetuximab *vs*. BSC	∅	572/4 *vs*. 6	PCR (sequencing)	1.8 *vs*. 3.0	NR	0 *vs*. 0	OS: 0.84 [NR-NR] PFS: 0.76 [NR-NR]	[42]
B**. Anti-VEGF therapies**									
*First-line therapy*									
**TRIBE**/III (R-SGA)	Bevacizumab; cf. two CTx backbones	FOLFOXIRI vs. FOLFIRI	508/16 *vs*. 12	Pyrosequencing	19.0 *vs*. 10.7	7.5 *vs*. 5.5	56 *vs*. 42	OS: 0.54 [0.24–1.20] PFS: 0.57 [0.27–1.23]	[28]
**Loupakis** *et al.*/II	Bevacizumab + CTx	FOLFOXIRI	25/25^**d**^	HRM analysis/sequencing	24.1	9.2	60	NR	[27]

*BSC* best supportive care, *CTx* chemotherapy, *EGFR* epidermal growth factor receptor, *HR* hazard ratio, *HRM* high-resolution melting, *NR* not reported, *ORR* overall response rate, *OS* overall survival, *PCR* polymerase chain reaction, *PFS* progression-free survival, *R‑SGA* retrospective subgroup analysis, *vs*. versus, *VEGF* vascular endothelial growth factor, *HR* hazard ratio, *CI* confidence interval

^a^Percentage of *BRAF*-mutant patients refers to total number of patients for whom results/tissue for *BRAF*^*V600E*^-mutation analysis were available (*BRAF*-mutant *versus BRAF *wild-type)

^b^According to Pietrantonio *et al.*, Eur J Cancer 2015 [24]

^c^Number of patients in the experimental study arm (i.e., panitumumab arm) with known *BRAF*^*V600E*^ mutation

^d^Validation cohort (*N* = 25) consisting of 15 patients prospectively included in this study and 10 patients from a previous study, in whom the BRAF status was retrospectively determined

Two partly overlapping meta-analyses confirmed a clinical benefit for anti-EGFR antibody therapy in patients with wild-type *RAS* and wild-type *BRAF*; in *RAS *wild-type patients harboring a *BRAF *mutation, however, data showed only a limited, non-significant clinical benefit in terms of progression-free survival (PFS) as well as OS [24, 25]. The current body of evidence, on the other hand, does not justify the exclusion of anti-EGFR antibodies from the therapeutic repertoire for *BRAF*^*V600E*^-mutant patients either. Data from the German randomized phase-II study VOLFI comparing panitumumab plus chemotherapy vs. mono-chemotherapy in first-line treatment showed that the addition of panitumumab to chemotherapy tended to increase the overall response rate (ORR) in the 14 patients with *BRAF*-mutated tumors (odds ratio = 14.93, 95% confidence interval [CI] 1.03–200.00) [26].

Anti-VEGF therapy, which—as with anti-EGFR therapy—is given in combination with oxaliplatin-containing (mostly in first-line) or irinotecan-containing chemotherapy (mostly in second-line), is a clinically relevant routine treatment of mCRC and can be used independent of *RAS *status. However, since no or only indirect comparisons are available to date, the predictive value of *BRAF *testing with regard to this treatment regimen is still unclear. Results of a small phase-II study [27] and subgroup analyses of two large phase-III studies [28, 29] also do not allow a clear overall assessment of the intensified chemotherapy backbone (FOLFOXIRI) in *BRAF*-mutant patients. A meta-analysis of five randomized studies of quite differing case numbers [range: 70–679] recently found that in *BRAF*-mutated patients—with the total case numbers still being small—intensified combination therapy does not provide an additional benefit in the first-line setting [30]. A meta-analysis performed by the ARCAD study group, published in autumn 2020 and pooling data from two studies comparing chemotherapy plus-anti-EGFR with chemotherapy plus-anti-VEGFR therapy as first-line options for mCRC, could not demonstrate a significant difference in OS for the subgroup of *BRAF*-mutated patients (*n* = 138) that received either bevacizumab-based or cetuximab-based therapy (HR = 1.01 [95% CI 0.69–1.48]) [31]. The benefit of anti-VEGF therapy with bevacizumab *per se* and the predictive role of *BRAF* for initiating bevacizumab-based therapy still requires further investigation (Table 1).

**Fig. 1:**
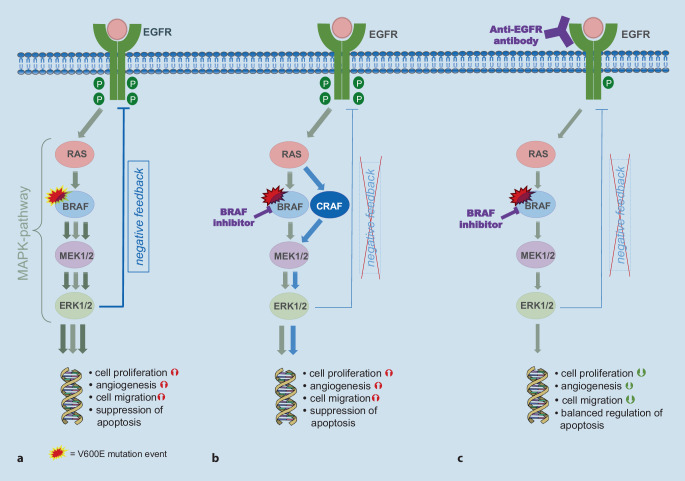
The MAPK signaling pathway (figure modified from Taieb et al. [15] CC BY licence). **a** MAPK pathway: signal enhancement in the presence of an activating *BRAF* mutation. **b** Inhibition of *BRAF* activated by mutation leading to suppression of the ERK-mediated negative feedback loop and reactivation of the MAPK signaling pathway via *CRAF*. **c** Counteracting resistance mechanisms: mechanism of action of combined *BRAF* and EGFR blockade. *BRAF* rapidly accelerated fibrosarcoma isoform B; *CRAF* rapidly accelerated fibrosarcoma isoform C; *EGFR* epidermal growth factor receptor; *ERK* extracellular signal-regulated kinase; *MAPK* mitogen-activated protein kinase; *MEK* MAPK/ERK kinase; *RAS* rat sarcoma protooncogene

Although targeted tyrosine kinase inhibitors have been used in clinical routine since 2011 with very good outcomes in *BRAF *^*V600*^-mutated melanoma, *BRAF*-mutated mCRC proved to be less sensitive to monotherapy [32, 33]. The reason behind this may be CRC-specific resistance mechanisms in the MAPK signaling cascade. *In-vitro* studies demonstrated suppression of the negative feedback loop between extracellular signal-regulated kinase (ERK) and the EGFR under BRAF monotherapy with overall high EGFR expression in CRC and possibly even stronger activation of the receptor by its ligands (Fig. 1; [33, 34]). This results in a reactivation of the EGFR pathway, e.g., by-passing the mutated *BRAF *protein via *CRAF*. Thus, it seems important to inhibit the EGFR pathway by simultaneously administering a therapy directed against EGFR in addition to *BRAF *blockade in order to block the multi-track resistance mechanisms within the MAPK signaling pathway [33, 35].

## Therapeutic options in *BRAF*-mutant mCRC

Until recently, first- and second-line treatment of mCRC has generally been based on the use of combination chemotherapies, mostly including—in the case of left-sided RAS wild-type tumors—EGFR antibody therapy, as described in the previous section [3, 4]^1^. Current treatment recommendations for mCRC are drawing much attention to the general condition of the patients, which are typically of older age. Regarding patients fit enough for systemic treatment, a distinction is made between the therapeutic objectives of “cytoreduction,” i.e., reduction of the tumor mass, and “disease control,” i.e., delaying further progression.

### First-line therapy in *BRAF*-mutant mCRC: a controversial treatment standard

The combination of an antimetabolite (5-fluorouracil, plus leucovorin as a folinic acid derivative) and a platinum compound interfering with DNA replication (oxaliplatin) together with a topoisomerase I inhibitor (irinotecan) and the angiogenesis inhibitor (anti-VEGF) bevacizumab represents the current European guideline standard for *BRAF*-mutant mCRC patients in good general health [4]. However, considering the evidence level in the *BRAF*-mutated subgroup, it has to be noted that this recommendation is based on a very small number of patients (*N* = 28) from the TRIBE study and is therefore associated with uncertainties [28]. In the *BRAF* subgroup of this phase-III study, the OS under FOLFOXIRI plus bevacizumab was 19 months with an ORR of 56%; however, there was no significant difference to the comparator group consisting of FOLFIRI plus bevacizumab (Table 1; [28]). This first-line standard is presently the subject of controversial discussion. A recently published meta-analysis based on five randomized studies on FOLFOXIRI plus bevacizumab vs. doublet chemotherapy plus bevacizumab showed a non-significant trend in *BRAF*-mutant tumors favoring the less intensive regime (*n* = 115; HR = 1.12 [95% CI 0.75–1.68]) [30].

Due to their poor prognosis, the concept of an ‘aggressive’ first-line treatment in *BRAF*^*V600E*^ -mutated mCRC, using almost the entire arsenal of therapy modalities, is currently of clinical relevance with regard to the current recommendations for this mCRC patient collective—especially when cytoreduction is the therapeutic objective. On the other hand, it is unclear to what extent patients in Germany are treated with this intensified first-line therapy, which is associated with relevant adverse events (AE).

As mentioned above, the issue of using anti-EGFR-based therapy in *BRAF*-mutant tumors is currently the subject of controversial debates due to the inconclusive results of two meta-analyses [24, 25].

### New chemotherapy-free, targeted option after systemic therapy

Due to the limited therapeutic options after completion of first-line treatment, no clear recommendations could be drawn to date for second- and third-line therapy of *BRAF-*mutant mCRC patients [3, 4]. The German S3 guideline stated in 2019 that “*Individual (presently) not approved therapeutic approaches, e.g. with a BRAF inhibitor, MEK inhibitor and anti-EGFR antibody or, if possible, treatment within a clinical study (are) to be taken into consideration*”; until recently, none of the therapeutic options mentioned here was approved for this indication.

In June 2020, however, the combination of the *BRAF* inhibitor encorafenib and the anti-EGFR antibody cetuximab was granted European Union (EU) approval, making such a chemotherapy-free, purely targeted dual blockade available for routine care. This combination is indicated for the treatment of adult patients with mCRC and a *BRAF*^*V600E*^ mutation who have received prior systemic treatment [43].

**Fig. 2:**
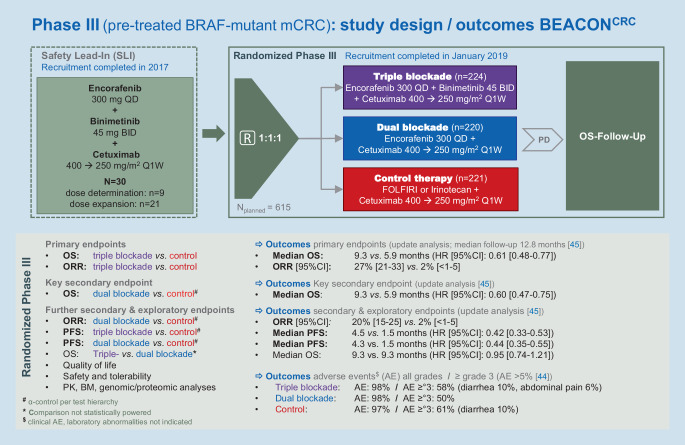
Design of the BEACON^CRC^ phase-III study in patients with pretreated metastatic colorectal cancer (*mCRC*) and *BRAF*^*V600E*^ mutation. *BID* twice daily; *BM* biomarker; *FOLFIRI* folic acid + fluorouracil + irinotecan; *ORR* overall response rate; *OS* overall survival; *PD* progressive disease; *PFS* progression-free survival; *PK* pharmacokinetics; *Q1W* weekly; *QD* once daily; *R* randomization; ° grade

The phase-III BEACON CRC trial, on which the approval was based, investigated the triple blockade with encorafenib and cetuximab plus the MEK inhibitor binimetinib and the dual blockade with encorafenib and cetuximab vs. control therapy consisting of irinotecan-based chemotherapy plus cetuximab in patients with *BRAF*^*V600E*^-mutant mCRC having previously received one or two palliative therapy lines (Fig. 2; [44]). Primary endpoints were ORR and OS of the triple blockade vs. the control. The study was powered on the key secondary endpoint: OS of dual blockade versus control. Using a test hierarchy, the secondary endpoints OS, ORR, and PFS of dual blockade vs. control and PFS of triple blockade vs. control were also alpha-controlled and thus confirmatory [44]. A total of 665 patients were randomized in a 1:1:1 ratio to the three therapy arms.

The primary endpoints of the study were reached. In the primary analysis (median follow-up for OS: 7.8 months), the combination of encorafenib, cetuximab, and binimetinib showed an ORR of 26% [95%CI 18–35] vs. 2% [95%CI < 1–7] in the control arm (*p* < 0.001). Median OS of the triple blockade vs. control was 9.0 vs. 5.4 months (HR = 0.52 [95% CI 0.39–0.70]; *p* < 0.001) [44]. For the alpha-controlled key secondary endpoint—the confirmatory comparison of OS of the dual combination therapy vs. control—an extension of the median OS by 3 months (8.4 vs. 5.4 months; HR = 0.60 [95% CI 0.45–0.79], *p* < 0.001) could be demonstrated for encorafenib plus cetuximab vs. the control group; the ORR was 20% [95%CI 13–29] vs. 2% [95%CI < 1–7] (*p* < 0.001).

The tolerability of the dual blockade was slightly more favorable than that of the triple blockade and of the control group (Fig. 2). The safety profile of the combination of encorafenib and cetuximab was well manageable and showed, in terms of AE, the expected class effects. The most common AEs included: elevated creatinine level (50%), nausea (34%), diarrhea (33%), low hemoglobin level (32%), fatigue (30%), acneiform dermatitis (29%), and decreased appetite (27%) [44].

Due to the comparable efficacy results of triple versus dual blockade and the slightly more favorable tolerability of encorafenib plus cetuximab, the European Medicines Agency (EMA) approved the dual combination regimen in June 2020 [43].

A new update analysis after a median follow-up of 12.8 months confirmed the above-described results with a consistent tolerability profile [45]. Median OS was 9.3 months [95% CI 8.2–10.8] for the triplet blockade, 9.3 months [95% CI 8.0–11.3] for the EMA-approved double blockade of encorafenib and cetuximab, and 5.9 months [95% CI 5.1–7.1] for the control group.

**Fig. 3:**
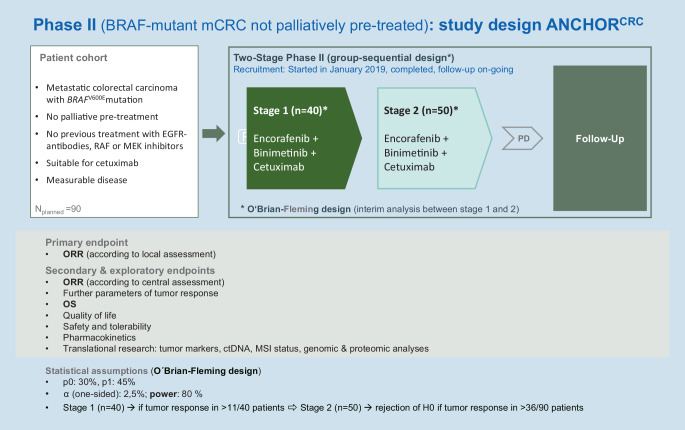
Design of the ANCHOR phase-II study in patients with previously untreated mCRC and *BRAF*^*V600E*^ mutation. *ORR* overall response rate; *OS* overall survival; *PD* progressive disease

The targeted triple blockade consisting of encorafenib, cetuximab, and binimetinib is currently being further investigated as a first-line therapy in the two-stage ANCHOR CRC phase-II study (Fig. 3).

## *BRAF* diagnostics

As a consequence of the above-described clinical situation, diagnostic testing for the presence of *RAS* and *BRAF *mutations in mCRC is essential: for *BRAF*-mutated patients, encorafenib plus cetuximab can be taken into consideration as a newly available therapeutic option.

**Fig. 4:**
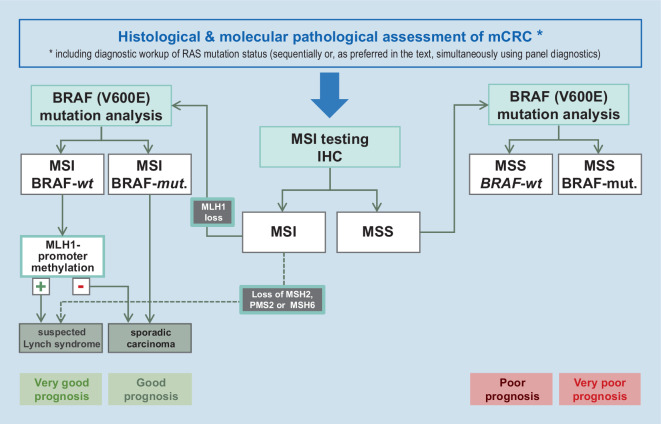
Diagnostic algorithm MSI/MSS—BRAF—MLH1 promoter methylation (at/after exclusion of a *RAS* mutation [3]; prognostic statements according to Lochhead *et* *al.* [17]). *BRAF* rapidly accelerated fibrosarcoma isoform B; *MLH1, MSH2, MSH6, PMS2* DNA repair enzymes/complexes; *MSI* microsatellite instability; *MSS* microsatellite stability; *wt* wild-type

The guidelines recommend performing these tests either before initiating first-line therapy in mCRC, or already at the time of initial diagnosis of CRC, in order to exclude the presence of Lynch syndrome via additional dMMR testing [3, 4]. In the same way as *BRAF*-mutated tumors, such hereditary CRCs without polyposis (HNPCC) also constitute a biologically distinct subtype of CRC. If a *BRAF* mutation is present in a dMMR/MSI tumor, Lynch syndrome can be mostly excluded. Thus, the determination of *BRAF *mutation status is of diagnostic and therapeutic relevance and helps to differentiate somatic from genetic “mismatch” repair defects [46, 47, 48]. The diagnosis of a sporadic tumor, thus excluding HNPCC/Lynch syndrome, can be supported by analyzing *MLH1 *promoter methylation, since the presence of such methylation additionally corroborates the diagnosis of a sporadic, high MSI (Fig. 4). *BRAF* testing can be performed either simultaneously with RAS testing or stepwise after excluding a *RAS *mutation. Nowadays, however, the simultaneous approach is recommended applying gene panel diagnostics based on focused next-generation sequencing (NGS).

### Sample preparation

In the course of the diagnostic workup, specimens are mostly obtained during colonoscopy or surgical removal of the primary tumor. After fixation via 10% neutral buffered formalin (4% formaldehyde) for 24–48 h and embedding in paraffin, the specimens are well suited for the tests indicated in Fig. 4. For molecular determination of MSI status, healthy tissue samples distant from the tumor should also be stored and analyzed [4]. If tissue samples contain a low percentage of tumor cells, a macro-dissection prior to DNA extraction is highly recommended for cancer cell enrichment.

### Molecular pathological, DNA analytical methods

Various test methods with different specificity and sensitivity are available to determine *BRAF*^*V600E*^ mutation status. With classical test methods (Table 2) such as Sanger sequencing, 99% of all mutations can be detected with a specificity of 100%. However, caution is required to ensure that the tumor cell proportion remains above 20–30%. Detection of the *BRAF*^*V600E*^ mutation by means of high-resolution melting (HRM) analysis or pyrosequencing provides higher sensitivity than Sanger sequencing; here, a tumor cell proportion of about 10–15% (5% detection limit) is sufficient [49, 50]. Commercial tests such as the ThxID-BRAF-Kit® (*V600E* and *V600K), *the cobas 4800® *BRAF* mutation test (only *V600E*), or the Idylla BRAF mutation test are of similar sensitivity (Table 3; [51, 52, 53]).

**Table 2:** Characteristics of classic and new DNA analytical methods for *BRAF* mutations

	Sanger	Pyrosequencing	HRM	NGS
Diagnostic type	Laboratory-based	Laboratory-based	Laboratory-based	Laboratory-based
Market approval^a^	Not required	Not required	Not required	EU: no/USA: partly yes
Indication	Multiple	Multiple	Multiple	Multiple
Selectivity^b^	Yes	Yes (Codon 600)	Yes	Yes
Specificity	100%	90%	100%	100%
Sensitivity	92%	> 98%	98–100%	≈ 100%
Limit of detection	10%	5%	6%	1/5%
In-lab time *(turnaround)*	2–3 Days	≈ 2 Days	≈ 1 Day	2–4 Days

*HRM* high-resolution melting; *NGS* next-generation sequencing

^a^In terms of CE label (EU) or *Pre-Market Approval* (USA)

^b^In terms of rare *BRAF* mutations (non-*BRAF*^V600E^)

**Table 3:** Characteristics of commercial test methods for analyzing *BRAF *mutations

	THxID® BRAF Kit	Cobas® 4800 BRAF V5600 mutation test	Idylla™ BRAF mutation test	Qiagen *therascreen*® BRAF V600E RGQ PCR kit	Foundation One® CDx
Diagnostic type	Standardized	Standardized	Standardized	Standardized	NGS
Market approval	USA (CDx), EU (CE)	USA (CDx), EU (CE)	USA (CDx), EU (CE)	USA (CDx), EU (CE)	USA (CDDx)
FDA PMA No. (year)	*P120014* (2012)	*P110020/S016* (2016)	(510(k) notification not required)	*P190026* (2020)	*P170019* (2017)
Indication	Melanoma	Melanoma	Multiple tumour indications	**CRC**	Multiple mutations und indications
Selectivity	Only V600E, V600K	V600E only	V600E/E2/D und V600K/R/M	V600E only	Only V600E, V600K
Sensitivity	> 96% ^V600E^; > 92% ^V600K^	> 98%	> 98%	> 98%	100%
Specificity	100%	> 98%	> 98%	100%	≈ 100%
Limit of detection	5% ^V600E^, 5% ^V600K^	5–7% ^V600E^, > 35% ^V600K^	Not specified	8%	2%
In-lab time (t*urnaround*)	1 day	1 day	2–4 h	1 day	≈ 3–5 days

*FDA PMA* Food and Drug Administration Premarket Approval, *CRC* colorectal cancer, *CDx* companion diagnostic; *CDDx* companion and/or complementary diagnostic; *NGS* next-generation sequencing

In recent years, NGS methods have been increasingly used in molecular diagnostics, allowing the detection of selected genes/genomic regions relevant for diagnosis and therapy (*targeted NGS*) simultaneously with high sensitivity and specificity. Therefore, *BRAF *mutation testing in CRC is often no longer carried out as an isolated individual test but integrated into the parallel detection of other molecular alterations such as *KRAS* and *NRAS*. The sensitivity of NGS-based methods is generally very high (approximately 1% detection limit), but hampered by artifacts occurring during formalin fixation. In many laboratories, a threshold of 5% variant allele frequency (VAF) is therefore requested, which can however be undercut in specific situations [49, 50]. Today, ThermoFisher Scientific and Illumina are the most prominent NGS platforms available. Both platforms enable analysis of numerous commercial or in-house gene panels, based on either amplicon-based (multiplex polymerase chain reaction, PCR) or hybrid-capture methods for enriching the selected target regions. Numerous bioinformatic programs are available for the evaluation of NGS data. However, these should be used by scientists/physicians that have profound experience in molecular diagnostics.

In the BEACON CRC study, which was conducted in a total of 221 centers in 28 countries (111 of which were centers in Europe), evaluation of the procedures used for *BRAF* status determination from 510 samples revealed the following picture: in 48.8% of the analyses, single gene detection was still used for *BRAF* testing; protein-based methods (immunohistology) were used in 0.7% of the analyses. However, the majority of *BRAF* tests were performed together with the detection of other gene alterations (e.g., as focused, amplicon-based NGS) (50.5%). Discrepancies observed between local and central testing show the relevance of standardization of diagnostic procedures, especially in view of the increasing importance of targeted therapeutic approaches: clear confirmation of the locally detected *BRAF*^*V600E*^ mutation was found in just 90.7% of central testing. Of note, this discrepancy was largely due to insufficient neoplastic tissue in the sample, most likely resulting from the fact that *BRAF*-mutant tumors are generally associated with mucinous adenocarcinoma that contain fewer tumor cells. In 1.6% of the central repeat tests, the local result was clearly negated. Taking into account this possibility of a discrepancy between local and central testing, the study protocol allowed the inclusion of patients based on local *BRAF*^*V600E*^ detection in molecular prescreening, but additionally required central confirmation, as part of the inclusion criteria, within 30 days of initial receipt of study medication. Once the study had reached the pre-specified number of discrepant test results, the assay, which was developed and then approved in the USA as a “companion diagnostic,” became a prerequisite for the inclusion of all further patients.

The EU and US regulations on in-vitro diagnostics (IVD) are fundamentally different: in the US, such tests are subject to central approval (Premarket Approval) by the Food and Drug Administration (FDA), while in the EU manufacturers can chose an accredited “notified body” that evaluates the conformity of their test; once confirmed, the manufacturer is allowed to label its product with the so-called CE label (CE: *Conformité Européene*)^2^. Furthermore, the FDA usually grants approval for targeted therapies only in conjunction with a defined and simultaneously approved *companion diagnostic,* which, at the time of drug approval, has an exclusivity of use: the usage of the respective companion diagnostic is therefore a prerequisite in the USA for the prescription of the drug by the physician [54].

The US legislation distinguishes such standardized, mostly commercially available tests from so-called* laboratory-developed tests* (LDT), which—like the classical test methods such as Sanger sequencing—are designed, validated, and applied by institutes for their own use. The German Accreditation Body (DAkkS) refers to such LDTs as “*in-house tests.*” They are normally not subject to formal approval or labeling requirements; however, a few years ago, the DAkkS issued a guideline for the validation of molecular pathological examination methods [55, 56]. In the US, a discussion paper was presented by the FDA in January 2017, advocating stronger prospective regulation of LDTs due to their increasing prognostic and predictive importance—this applies in particular to the role and growing importance of NGS [57, 58]. With the FoundationOne® CDx (F1CDx) test, an NGS method was approved as a *companion diagnostic* for the first time in the USA in late 2017 (Table 3; [59]).

Against this background, the diversity of competing classical and modern DNA analytical methods for *BRAF* mutation determination is easier to understand. Of the various commercial procedures using allele-specific PCR techniques, only the Qiagen *therascreen*® test is currently recommended for *BRAF-*mutated mCRC in the US; in Europe, this test is CE-labeled. It is to be expected that for the tests currently approved for melanoma only, appropriate adjustments will soon be made in the US with regard to CRC. In the BEACON CRC approval study, the only methods allowed by the study protocol were PCR and NGS based on local assays [44].

A comparison between commercially available (i.e., FDA-approved) tests and LDTs for *EGFR, KRAS*, and *BRAF *mutations showed that there was no overall difference between the methods and the three tested genes in assay performance; the average analysis accuracy was 97% [60]. Since testing for *KRAS, BRAF, MSI*/*dMMR, MLH1*, and possibly other genes constitutes a prerequisite of CRC diagnostics, panel-based assays are understandably more prominent in current pathological practice—in Germany, all major university and non-university institutions are now using focused NGS for this purpose. Frequently used platforms include Illumina (MiSeq™ or NextSeq™) and Thermo Fisher (Ion Gene Studio S5™) [61, 62, 63]: in a multicenter validation study across Germany, a high level of consistency between different NGS platforms and gene panels was shown; apart from CRC, samples of lung and breast cancers were also tested [61].

### Immunohistochemical methods

Besides DNA analytical methods, protein-based analyses using the VE1 antibody may provide an alternative to molecular pathological testing for *BRAF*^*V600E*^; the latter is widely regarded as the gold standard in *BRAF *mutation testing [50, 64]. At the same time, protein-based immunohistological detection is the only reasonably practicable method for determining the expression level of mutant *BRAF *protein. This method is also applicable for MSI testing. It is characterized by a specificity of 98–100%, a sensitivity of 85–100% [49, 50], and an in-lab turnaround time of 1 day. Thus, the method is generally reliable, but some challenges remain, such as establishing a standardized scoring of protein expression, which is needed to avoid a substantial number of misclassifications [64]. Immunostaining is in principle a fast and cost-effective method for the determination of *BRAF-*mutant protein; however, as CRC meanwhile requires the determination of multiple alterations, DNA analytical methods should certainly be preferred nowadays.

Chu et al. investigated outcomes of immunohistochemical (IHC) and NGS testing in a cohort of almost 1900 CRC patients [65]. The rate of false-positive IHC tests was 17%; however, confirmatory re-testing by NGS was performed in only 43% of the IHC-tested patients. NGS-tested patients had a favorable median OS, at younger age, and a lower rate of synchronous metastases and a higher rate of therapy. The authors concluded that NGS should be considered as standard testing but that IHC might serve as an optional screening test if NGS testing is not available in a timely manner. This is underlined by the rapid availability of results via IHC and the finding that reflexive IHC testing made it possible to identify 57% more *BRAF*-mutated mCRC than standard NGS methods.

## Conclusions for clinical practice

- The detection of a *BRAF*^*V600E*^ mutation, particularly in the presence of MSS status, is associated with a dismal prognosis, indicating an aggressive molecular subtype of CRC.
- For Patients with *BRAF*^*V600E*^-mutated CRC and their “high unmet medical need,” encorafenib plus cetuximab now constitutes a chemotherapy-free, targeted therapy standard after systemic treatment; combined *BRAF* and EGFR blockade is expected to be included in the German and European treatment algorithms and guidelines in the coming months.
- For adequate planning of the therapy sequence, *BRAF *testing is therefore an absolute necessity in all patients with mCRC before initiating first-line therapy.
- For testing, a large variety of methods is available, although panel diagnostics with *NGS* should be preferred to integrate testing for various molecular alterations.

## Footnotes

1 Regarding the guidelines of the European Society of Medical Oncology (ESMO), there are not only the *Consensus Guidelines*, latest edition published in 2016, for metastatic CRC (see Ref. [4]), but also the *Pan-Asian Adapted ESMO Consensus Guidelines* published in early 2018. Although this article refers to the somewhat older ESMO guideline, the reference always applies to both guidelines and the current study evidence shown therein.

2 With the “CE label,” the manufacturer, distributor, or EU authorized representative declares under EU Regulation 765/2008 “that the product meets the applicable requirements stipulated in the harmonization legislation of the Community on its affixing.”

## Declarations

For this article no studies with human participants or animals were performed by any of the authors. All studies performed were in accordance with the ethical standards indicated in each case.

The supplement containing this article is not sponsored by industry.
